# Mobilizing resources with an investment case to mitigate cross-border malaria transmission and achieve malaria elimination in South Africa

**DOI:** 10.1080/16549716.2023.2205700

**Published:** 2023-05-09

**Authors:** Aparna Kollipara, Devanand Moonasar, Ryleen Balawanth, Sheetal P. Silal, Anthony Yuen, Katie Fox, Joseph Njau, Yogan G. Pillay, Mark Blecher

**Affiliations:** a San Francisco Global Health Group, Malaria Elimination Initiative at the University of California, San Francisco, CA, USA; b National Department of Health, Malaria Vector and Zoonotic Disease Directorate, Pretoria, South Africa; c School of Public Health and Health Systems, University of Pretoria, Pretoria, South Africa; d South Africa Regional Office, Clinton Health Access Initiative, Inc. (CHAI), Pretoria, South Africa; e Modelling and Simulation Hub, Africa (MASHA), Department of Statistical Sciences, University of Cape Town, Cape Town, South Africa; f Centre for Tropical Medicine and Global Health, Nuffield Department of Medicine, Oxford University, Oxford, UK; g JoDon Consulting Group, Health Economist, Lilburn, GA, USA; h Affiliate Center for Innovation in Global Health, Georgetown University, Washington, DC, USA; i Public Finance Division, National Treasury, Pretoria, South Africa

**Keywords:** Resource mobilisation, malaria financing, co-financing, return on investment, cross-border collaboration

## Abstract

South Africa’s effort to eliminate malaria is significantly challenged by a large number of imported malaria cases, especially from neighbouring Mozambique. The country has a funding gap to achieve its malaria elimination goals (prior to 2019) and is ineligible to receive a national allocation from the Global Fund. The findings of an IC were utilised to successfully mobilise resources for malaria elimination in South Africa in 2018. A five-step resource mobilisation strategy was implemented to highlight financing challenges and leverage the economic evidence from an IC for malaria elimination in South Africa. South Africa’s malaria programme implements control and elimination activities in three malaria-endemic provinces (KwaZulu Natal, Limpopo, and Mpumalanga). Driven by the IC findings, the South African government took an unprecedented step and increased total domestic malaria financing by approximately 36%, from the 2018/19 to the 2019/20 financial years through the creation of a new conditional grant for malaria. The IC findings predicted that malaria control in southern Mozambique is a prerequisite to eliminate malaria in South Africa. Based on this, the South African government also allocated funding towards a co-financing mechanism to support malaria control efforts in southern Mozambique. The IC findings assisted the South African National Department of Health to make a convincing case to key government decision-makers to invest in national malaria elimination and maximise economic returns in the long run. The South African government is the first in Southern Africa to mobilise a significant increase in domestic malaria financing to address the financial sustainability of both national and regional malaria elimination efforts. Continued surveillance activities will be required to prevent the re-establishment of malaria transmission even after malaria elimination is achieved in South Africa. Information sharing and close collaboration with provincial and national government officials were key to the successful outcome.

## Background

The 2012–2018 National Malaria Elimination Strategic Plan set the malaria elimination goal in South Africa (SA) for 2018. However, in 2017, SA experienced a malaria outbreak that forced the country to reassess the financial feasibility of achieving national elimination. From the 2008/9 to 2018/19 financial years, the domestic malaria budget had seen low nominal growth [1]. Experts argue that reduced financial or political commitments in endemic countries have historically been associated with an increased risk for malaria resurgence [2]. Since the 1930s, 91% of 75 malaria resurgence events in 61 countries were attributed to the weakening of malaria control programmes, primarily due to resource constraints [2,3]. Despite stagnant domestic malaria financing trends, SA has made significant progress in malaria control in the past 20 years [4]. However, the 2017 malaria outbreak led to a dramatic increase in malaria hospitalisations which demonstrated that adequate malaria financing to prevent outbreaks is of paramount importance to achieve malaria elimination.

**Figure 1.**
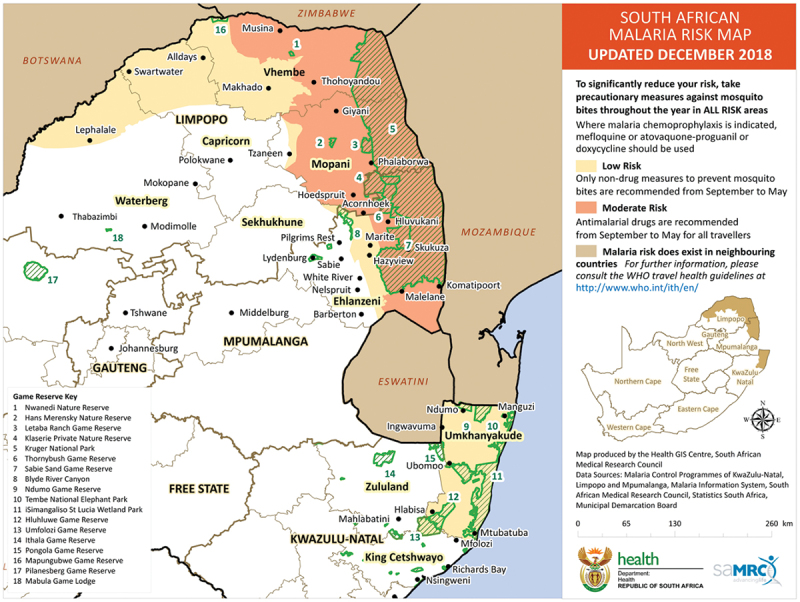
South African malaria risk map 2018 [7].

Malaria is deprioritised against high burden diseases (e.g. HIV and TB) alongside declining trends in donor support [3,5,6]. As a result, the South African malaria programme has historically struggled to mobilise domestic and external resources for malaria elimination. Figure 1 depicts SA as a low-transmission country which shares borders with two high-transmission countries (Zimbabwe and Mozambique) [7]. The feasibility of achieving malaria elimination in SA and the Southern African region, among others, rests on the region’s ability to finance interventions that address cross-border transmission. Malaria cases in South Africa are imported mainly from southern Mozambique, hence co-financing was an important option to pursue [8].

This paper highlights SA’s success story in mobilising domestic resources for malaria elimination, the first of its kind in the Southern African Development Community (SADC) region [8].

## Local setting

Unlike most countries in sub-Saharan Africa, South Africa’s malaria response is funded domestically, except for regional, cross-border initiatives funded by the Global Fund to Fight AIDS, TB, and Malaria through the Elimination 8 (E8) and Mozambique, South Africa, and Eswatini (MOSASWA) initiatives [9]. The E8 and MOSASWA are cross-border and regional initiatives that receive regional funding from the Global Fund. SA is ineligible to receive a national allocation from the Global Fund due to its upper middle-income status and low malaria disease burden, despite the funding gap to address the additional burden of imported cases from neighbouring countries [10,11]. The SA malaria programme is funded from two domestic sources: (1) funding allocated to the National Malaria Program of the National Department of Health (NDOH) which sets national malaria policy and guidelines; and (2) funding allocated to the Limpopo, Mpumalanga, and KwaZulu-Natal provincial malaria programmes through what is called Provincial Equitable Share (PES), an unconditional budget allocation to the provinces.

## Approach: resource mobilization strategy to advance the IC findings

The resource mobilisation strategy was a five-part process that was conducted over the course of a year (2018–2019) that entailed close involvement with country-level stakeholders and government decision-makers on the investment case (IC) [12,13].

**Table 1.** The five-step resource mobilisation strategy is outlined in Table 1.

Step in Resource Mobilization Strategy	Methods	Outcomes
1. Identify the problem February 2018–May 2018	Key informant interviews were conducted with national and provincial malaria officials to collect information on the current malaria response, financing trends, and the challenges to achieving national malaria elimination and mobilising resources towards the response.	The key informant interviews provided insight into how to address the challenges to fund malaria elimination in SA and the possible avenues to advocate for funding. This information served as the foundation of the resource mobilisation strategy. These meetings facilitated collaboration among government stakeholders on the malaria elimination goal.
2. Identify key stakeholders March 2018–June 2018	The IC Technical Task Team (TTT) was formed at the beginning of the IC study comprising members from NDOH, research institutions, and partner organizations. The full list of TTT members is in the Appendix.	The TTT informed the development of the strategy (e.g. budget request and financing mechanisms), offering advice on interventions to include in the IC study and to fund to achieve the elimination goal.
3. Generate economic evidence through the IC study February 2018–August 2018	Using a malaria transmission model, a cost–benefit analysis of malaria elimination was conducted through simulating three scenarios to achieve malaria elimination.	Findings predicted that national malaria elimination is achievable by 2026 and is good value for money, with a national return on investment (ROI) of US$4.2 realised in SA plus an additional ROI of US$3.01 realised in southern Mozambique.
4. Submit a budget request informed by the IC findings August 2018	The NDOH, with technical support from the TTT, presented a malaria budget request to the South African National Treasury [8].	NDOH successfully mobilised approximately US$ 21M for the three-year 2019 Medium Term Expenditure Framework (MTEF) period, which equated to a 36% increase from the 2018/19 malaria budget. This is recurring funding that is renewed annually.
5. Mobilise additional malaria financing and create new financing mechanisms August 2018−April 2019	The TTT engaged with multiple stakeholders to determine the most suitable financing mechanisms.	The South African government provided approximately US$ 4.1M for malaria control efforts in southern Mozambique.

Step 1: The IC research team conducted qualitative key informant interviews with national and provincial government officials to map the current malaria response efforts and the financing landscape, and to record the main programmatic challenges to attaining national malaria elimination. These conversations created awareness about the challenges among key government officials and provided key insight into the potential opportunities to secure additional financing for malaria elimination.

Step 2: Resource mobilisation efforts were coordinated through the IC technical task team (TTT), which was established by the National Malaria Program Director to guide the design and implementation of the resource mobilisation strategy. The TTT represents the South African Malaria Elimination Committee (SAMEC), government, and partner organisations with the unified goal of mobilising resources for malaria elimination. The TTT members included epidemiologists, economists, and mathematical modellers who offered technical insight into the IC. TTT played an important role in building country-level buy-in and consensus at critical stages of resource mobilisation strategies such as the development of budget requests and the creation of new malaria financing mechanisms.

Monthly budget meetings with key health and finance officials were held to raise a spotlight on malaria, to present the findings of the IC, seek feedback, and ultimately to influence government’s decision to commit additional funding towards the elimination goal.

Step 3: An IC was conducted to estimate the malaria financing gap, ROI of elimination, the direct costs averted to the health system and households, and indirect costs averted to society due to malaria morbidity that were used to inform the budget request (for full details, see [8]). A mathematical transmission model was used to simulate three scenarios (*Business as Usual, Accelerate* and *Source Reduction)* aimed at achieving malaria elimination. The *Source Reduction* scenario modelled the scale-up of current malaria activities (*Accelerate* scenario) plus the implementation of source reduction activities in southern Mozambique which predicted that national malaria elimination is achievable by 2026 and is good value for money, with a national ROI of US$4.2 realised in SA plus an additional ROI of US$3.01 realised in southern Mozambique [8]. These findings were used to build a case for additional malaria financing in the form of a budget request from NDOH to the National Treasury.

Step 4: The TTT provided technical support to the NDOH to develop the malaria budget request that included the problem statement, malaria financing trends from domestic and external sources, the rationale for additional malaria financing, the funding requirements for the *Accelerate* and the *Source reduction* scenarios, and the cost-benefit analysis from the IC [14]. The budget request also presented two potential financing mechanisms for malaria, a direct conditional grant earmarked for malaria and a co-financing mechanism for malaria control in southern Mozambique.

## Relevant changes: new financing mechanisms created

Step 5: The South African Government approved NDOH’s request to fully fund source reduction per the *Source Reduction* scenario and created two new financing mechanisms for malaria. Conditional grant and co-financing allocations were made as recurring, not one-time, allocations to the budget baseline.

The new, ring-fenced malaria conditional grant component serves as a supplement to existing malaria financing in PES. NDOH transfers the conditional grant funding as monthly disbursements to KwaZulu-Natal, Limpopo, and Mpumalanga provinces. The conditions and performance indicators of the conditional grant framework for the malaria component were informed by the IC findings and inputs from the TTT and provincial malaria programme managers (in Table 2) [15].

**Table 2.** Malaria conditional grant performance indicators.

**Conditions**	**The following priority areas must be supported through the grant**: Malaria surveillance, prevention, treatment Mobile active testing units Testing and treating through active testing in the community Malaria vector control Indoor residual spraying Integrated vector management activities Programme management strengthening for malaria elimination Hiring of staff for approved malaria posts
**Performance Indicators**	Number of malaria-endemic municipalities with >95% indoor residual spray (IRS) coverage Percentage confirmed cases notified within 24 h of diagnosis Percentage of confirmed cases investigated and classified within 72 h Percentage of identified health facilities with recommended treatment in stock Percentage of identified health workers trained on malaria elimination Percentage of population reached through malaria information education and communication (IEC) on malaria prevention and early health-seeking behaviour interventions Percentage of vacant funded malaria positions filled Number of malaria camps refurbished and/or constructed

The creation of this financing mechanism has, in effect, elevated malaria elimination as a national priority. The conditional grant has addressed decades-long financial gaps that have previously hampered malaria elimination progress. Key government leaders recognised that investing in malaria elimination is a catalyst for health and broader economic gains in SA and the southern African region.

The TTT’s advocacy efforts for source reduction in southern Mozambique as a key intervention for national malaria elimination led the South African government to approve the creation of a co-financing mechanism, a novel funding approach to regional elimination in which a country’s government provides funding support to a neighbouring country’s malaria effort. The two financing mechanisms (USD 27 million over the 2019 Medium Term Expenditure Framework period^1^^1^2019 Medium Term Expenditure Framework period is 2019/20 to 2011/2022 financial years.) were built into the budget baseline and were a clear demonstration of the South African government’s sustained commitment to the elimination goal [3,12].

## Lessons learnt

The collaboration between national and provincial health and treasury officials was pivotal to securing funding for malaria. Regular updates on the IC study helped to keep malaria elimination on the agenda of key budget meetings between the NDOH and the National Treasury. Government decision-makers found the IC to be innovative (the methods applied both epidemiological and economic modelling techniques) and highly credible as it was informed by the varied and experienced members of the TTT (refer to Box 1 for a summary of the main lessons learnt).

Conditional grant indicators are measured on a quarterly basis to assess performance and identify implementation challenges. The increase in funding came with additional responsibilities to cost expanded interventions and monitor spending of the new malaria funding. Provincial malaria programmes lacked the requisite project and financial management skills to conduct these tasks. With recruitment delays in the provinces, it will take time to build these skills within the provincial malaria programmes and improve conditional grant spending and performance [16].

**Box 1.** Summary of main lessons learnt.

Government officials that make long-term health financing decisions found the combined epidemiological and economic methods to analyse the costs and benefits of malaria elimination to be innovative and useful. A clearly documented budget request for malaria elimination was an effective tool to present the challenges to malaria elimination, a plan of action, funding requirements, and to propose financing options. The most challenging aspect was to finalise the budget request for malaria elimination within the deadlines of the South African Budget Process. It required close collaboration with the Department of Health and National Treasury to align on objectives and to collate the IC findings alongside inputs from multiple stakeholders. It was critical to continually engage national and provincial government officials during the budget process to provide information/data and respond to queries. Presenting at budget meetings where all key stakeholders were present proved to be the most effective mode of communication.

## Thoughts for the future

The success of cross-border collaboration to reduce malaria transmission has been demonstrated previously through the Lubombo Spatial Development 1 (LSDI1), which was implemented from 2000 to 2012 [17]. The South African government’s renewed commitment to regional elimination through co-financing source reduction activities in southern Mozambique will hopefully lead to future opportunities for improved collaboration in the SADC region’s malaria response [3]. Once elimination is achieved in endemic areas, continued surveillance measures to prevent the re-establishment of malaria transmission will be necessary in receptive areas as well as non-receptive areas (e.g. Gauteng province) [18].

Finally, it is important to note that existing external resources are inherently finite and funder priorities are likely to shift over time. The co-financing allocation made by the South African government is a step towards deepening regional cooperation for mutual gain. There are also a limited number of malaria-endemic countries that have the fiscal space, as demonstrated in SA, to reprioritize domestic funding towards malaria elimination. Therefore, opportunities must be explored to mobilise funding from other sources, such as private sector partners that would benefit from malaria elimination [3].

## Conclusions

A five-step resource mobilisation strategy was implemented to highlight financing challenges and leverage the economic evidence from an IC for malaria elimination in SA. NDOH, with guidance from the TTT, successfully executed the resource mobilisation strategy that led to increased investments towards national malaria elimination to maximise long-term economic returns. In January 2023, SA received a global award from the World Health Organization for successfully mobilising domestic resources for malaria elimination [19]. This is a rare achievement for regional cooperation or South–South cooperation; the South African government not only substantially increased malaria financing for domestic response efforts but also took the bold step to fund case reduction in a neighbouring country where transmission is significantly higher. Saudi Arabia has demonstrated a similar cross-border collaboration with neighbouring Yemen with the aim of regional malaria elimination [20]. However, poor geopolitical relations or misaligned surveillance strategies pose as barriers to a successful cross-border collaboration [21]. Other higher GDP countries should consider an IC approach to mobilise resources for domestic and regional malaria elimination. A regional approach is predicted to achieve a greater ROI compared to national investment alone [22].

## Paper context

This paper details a five-step resource mobilisation strategy for malaria elimination implemented in South Africa. The strategy facilitated the South African government’s decision to create two domestic financing mechanisms for malaria: (1) a conditional grant and (2) a co-financing mechanism for malaria source reduction activities in southern Mozambique. Government decision-makers recognised that a significant increase in malaria financing now will yield a return on investment through the reduction of malaria-associated morbidity and mortality.

## APPENDIX

### List of South African investment case technical task team members

1. Mr. Aaron Mabuza, Clinton Health Access Initiative
2. Mr. Anthony Yuen, Regional Financing Associate – Clinton Health Access Initiative
3. Ms. Aparna Kollipara, Senior Programme Manager – UC San Francisco
4. Prof. Basil Brooke, Head Vector Control – Division of the Centre for Emerging, Zoonotic & Parasitic Diseases – NICD
5. Mr. Bheki Qwabe, KwaZulu-Natal Department of Health
6. Ms. Bridget Mbavhalelo Shandukani, Assistant Director: Malaria, Other Vector-borne and Zoonotic Diseases Directorate – NDOH
7. Dr. Devanand Moonasar, Director: Malaria, Other Vector-borne and Zoonotic Diseases Directorate – NDOH
8. Mr. Eric Mabunda, Provincial Malaria Manager – Limpopo Department of Health
9. Mr. Eric Raswiswi, Malaria Provincial Manager – KwaZulu-Natal Department of Health
10. Dr. Eunice Misiani, Deputy Director: Malaria, Other Vector-borne and Zoonotic Diseases Directorate – NDOH
11. Ms. Gillian Malatje, Malaria Provincial Manager – Mpumalanga Department of Health
12. Mr. Hadley Nevhutalu, Chief Director, Provincial Financial Management Support – NDOH
13. Dr. Indongesit Sunday Ukpe – Mpumalanga Department of Health
14. Dr. Jaishree Raman, Medical Scientist – Parasitology Reference Laboratory – Division of the Centre for Emerging, Zoonotic & Parasitic Diseases, NICD
15. Mr. Jorge Quevedo, Country Director – Clinton Health Access Initiative
16. Dr Joseph Njau, Consultant to UC San Francisco
17. Prof. Karen Barnes, Head of the Pharmacology Group in WWARN and the lead for the Southern Africa Regional Centre – University of Cape Town
18. Ms. Katie Fox, Programme Coordinator − UC San Francisco
19. Prof Lucille Bloomberg, Deputy Director Epidemiology – Division of the Centre for Emerging, Zoonotic & Parasitic Diseases, NICD
20. Dr. Natalie Mayet, Deputy Director – National Institute of Communicable Diseases
21. Dr. Natashia Morris, GIS Specialist – SA Medical Research Council
22. Mr. Philip Kruger, Acting Chief Director of District Support Services – Limpopo Department of Health
23. Prof. Rajendra Maharaj, Director of MOMR – SA Medical Research Council
24. Ryleen Balawanth, Malaria Programme Manager – Clinton Health Access Initiative
25. Dr. Sheetal Silal, University of Cape Town, consulting to UC San Francisco

## References

[1] National Department of Health (NDOH), Provincial Department of Health, Medical Research Council, National Institute of Communicable Disease. Malaria programme budgets, financial years 2008/2009-2018/2019. Cape Town, South Africa: Department of Health, Government of South Africa; 2020.

[2] Cohen JM, Smith DL, Cotter C, Ward A, Yamey G, Sabot OJ, et al. Malaria resurgence: a systematic review and assessment of its causes. Malaria J. 2012;11:1–7. 10.1186/1475-2875-11-122 PMC3458906 22531245

[3] Organization WH. Global technical strategy for malaria 2016-2030, 2021 update. Geneva: World Health Organization; 2021.

[4] Republic of South Africa. National Malaria Information System (MIS). In: National Department of Health, editor. Gauteng, Pretoria: National Department of Health (NDOH); 2020.

[5] South African Development Cooperation (SADC). SADC malaria report 2017. South Africa: SADC, 2017.

[6] Blecher MS, Kollipara A, Daven J, Meyer-Rath G, Chiu C, Pillay Y, et al. HIV and AIDS financing in South Africa: sustainability and fiscal space. South Afr Health Rev. 2016;2016:203–219.

[7] National Department of Health. National Malaria Control Programme - malaria epidemiology and situation analysis. In: Department of Health, editor. Cape Town,South Africa: Government of South Africa; 2018.

[8] Njau J, Silal SP, Kollipara A, Fox K, Balawanth R, Yuen A, et al. Investment case for malaria elimination in South Africa: a financing model for resource mobilization to accelerate regional malaria elimination. Malaria J. 2021;20:1–16. 10.1186/s12936-021-03875-z PMC8365569 34399767

[9] Moonasar D, Maharaj R, Kunene S, Candrinho B, Saute F, Ntshalintshali N, et al. Towards malaria elimination in the MOSASWA (Mozambique, South Africa and Swaziland) region. Malaria J. 2016;15:419. 10.1186/s12936-016-1470-8 PMC4991067 27538990

[10] The Global Fund. The Global Fund Eligibility List. In: Global Fund, editor. Access to funding. Geneva: Global Fund; 2022. Available from: https://www.theglobalfund.org/media/11712/core_eligiblecountries2022_list_en.pdf

[11] Zelman B, Melgar M, Larson E, Phillips A, Shretta R. Global fund financing to the 34 malaria-eliminating countries under the new funding model 2014–2017: an analysis of national allocations and regional grants. Malaria J. 2016;15:118. 10.1186/s12936-016-1171-3 PMC4766696 26911998

[12] Cohen JM, Kandula D, Smith DL, Le Menach A. How long is the last mile? Evaluating successful malaria elimination trajectories. Malar J. 2022;21:330. Epub 20221114. PubMed PMID: 36376935; PubMed Central PMCID: PMC9664685. 36376935 10.1186/s12936-022-04368-3 PMC9664685

[13] World Health Organization. WHO technical brief for countries preparing malaria funding requests for the global fund (2020-2022). In: Organization WH, editor. Chapter 2: Use of Strategic Information to Drive Impact. Geneva: WHO; 2020.

[14] Sabot O, Cohen JM, Hsiang MS, Kahn JG, Basu S, Tang L, et al. Costs and financial feasibility of malaria elimination. Lancet. 2010;376:1604–1615. 21035839 10.1016/S0140-6736(10)61355-4 PMC3044845

[15] Republic of South Africa. Division of revenue act. In: Treasury Department, editor. Pretoria: Government of South Africa; 2019.

[16] Department of Health. Fourth quarter provicial performance report for the health conditional grants, 2019/20 financial year. In: National Department of Health, editor. Government of South Africa; 2020;1–14. Malaria Component Annual Report.

[17] Maharaj R, Moonasar D, Baltazar C, Kunene S, Morris N. Sustaining control: lessons from the Lubombo spatial development initiative in southern Africa. Malaria J. 2016;15:409. 10.1186/s12936-016-1453-9 PMC4983057 27520364

[18] Brooke BD. Malaria vector surveillance and control in an elimination setting in South Africa. Trop Med Infect Dis. 2022;7:391. PubMed PMID. 36422942 10.3390/tropicalmed7110391 PMC9698861

[19] WHO-South Africa. 2023. Working together towards malaria elimination. In: WHO Africa, editor. South Africa: WHO. Available from: https://www.afro.who.int/countries/south-africa/news/working-together-towards-malaria-elimination

[20] Coleman M, Al-Zahrani MH, Coleman M, Hemingway J, Omar A, Stanton MC, et al. A country on the verge of malaria elimination–the Kingdom of Saudi Arabia. PLoS One. 2014;9:e105980. Epub 2014/09/25. PubMed PMID: 25250619; PubMed Central PMCID: PMC4175080. 25250619 10.1371/journal.pone.0105980 PMC4175080

[21] Arisco NJ, Peterka C, Castro MC. Cross-border malaria in Northern Brazil. Malaria J. 2021;20:1–13. 10.1186/s12936-021-03668-4 PMC7937307 33676522

[22] Shretta R, Silal SP, Malm K, Mohammed W, Narh J, Piccinini D, et al. Estimating the risk of declining funding for malaria in Ghana: the case for continued investment in the malaria response. Malaria J. 2020;19:1–15. 10.1186/s12936-020-03267-9 PMC7268595 32487148

